# Histological assessment of nanostructured fibrin‐agarose skin substitutes grafted in burnt patients. A time‐course study

**DOI:** 10.1002/btm2.10572

**Published:** 2023-07-07

**Authors:** Miguel Angel Martin‐Piedra, Gloria Carmona, Fernando Campos, Víctor Carriel, Ana Fernández‐González, Antonio Campos, Natividad Cuende, Ingrid Garzón, Purificación Gacto, Miguel Alaminos

**Affiliations:** ^1^ Tissue Engineering Group, Department of Histology University of Granada Spain; ^2^ Instituto de Investigación Biosanitaria ibs.GRANADA Granada Spain; ^3^ Andalusian Network for the Design and Translation of Advanced Therapies (former Andalusian Initiative for Advanced Therapies) ‐ Fundación Andaluza Progreso y Salud, Junta de Andalucía, Seville, Spain; Andalusian Transplant Coordination, Servicio Andaluz de Salud Seville Spain; ^4^ Doctoral program in Biomedicine University of Granada Granada Spain; ^5^ Unidad de Producción Celular e Ingeniería Tisular Hospital Universitario Virgen de las Nieves Granada Spain; ^6^ Burn Unit, University Hospital Virgen del Rocío Seville Spain

**Keywords:** artificial skin, burnt patients, histology, tissue engineering

## Abstract

A previously developed fibrin‐agarose skin model—UGRSKIN—showed promising clinical results in severely burnt patients. To determine the histological parameters associated to the biocompatibility and therapeutic effects of this model, we carried out a comprehensive structural and ultrastructural study of UGRSKIN grafted in severely burnt patients after 3 months of follow‐up. The grafted epidermis was analogue to native human skin from day 30th onward, revealing well‐structured strata with well‐differentiated keratinocytes expressing CK5, CK8, CK10, claudin, plakoglobin, filaggrin, and involucrin in a similar way to controls, suggesting that the epidermis was able to mature and differentiate very early. Melanocytes and Langerhans cells were found from day 30th onward, together with a basement membrane, abundant hemidesmosomes and lack of rete ridges. At the dermal layer, we found an interface between the grafted skin and the host tissue at day 30th, which tended to disappear with time. The grafted superficial dermis showed a progressive increase in properly‐oriented collagen fibers, elastic fibers and proteoglycans, including decorin, similarly to control dermis at day 60‐90th of in vivo follow‐up. Blood vessels determined by CD31 and SMA expression were more abundant in grafted skin than controls, whereas lymphatic vessels were more abundant at day 90th. These results contribute to shed light on the histological parameters associated to biocompatibility and therapeutic effect of the UGRSKIN model grafted in patients and demonstrate that the bioengineered skin grafted in patients is able to mature and differentiate very early at the epithelial level and after 60–90 days at the dermal level.

## 1 INTRODUCTION

Large burns are very relevant public health problems causing massive tissue destruction and dramatic pathophysiologic effects on the burnt patient.^1^ As an intact healthy skin is necessary to protect the internal tissues, a proper skin barrier is essential for patient survival.^2^ However, treatment of these patients is challenging, since the severe and deep skin damage typically destroys the skin stem cells, and the damaged tissue is not able to regenerate by itself.^3^

Development of human artificial skin substitutes by tissue engineering offers a promising regenerative treatment for burnt patients.^4^, ^5^ In general, these skin substitutes consist of a scaffold biomaterial and skin cells. Although several skin substitutes have been developed, and promising clinical results were demonstrated for some of them,^6^, ^7^ an ideal substitute has not been described to the date. In this regard, we developed a bioartificial model of human skin called UGRSKIN or NANOGSKIN, which combines highly biocompatible fibrin‐agarose biomaterials containing human dermal fibroblasts with human skin keratinocytes able to form a stratified epithelial layer on top. This skin model was characterized ex vivo and in vivo in laboratory animals using several histological, histochemical, immunohistochemical, and other analysis methods able to demonstrate that the model was safe and efficient.^8^, ^9^, ^10^ In fact, our preclinical studies demonstrated that the biochemical properties and histological structure of the UGRSKIN skin model were similar to the native human skin at both, the epidermal and the dermal layers. Ex vivo, UGRSKIN showed to be biomechanically comparable to the native human skin, with adequate properties as determined by compressive and shear tests.^11^ Bioartificial tissues based on fibrin‐agarose biomaterials displayed an elastic behavior and its modulus of Young was comparable to native tissues,^11^ and our previous time‐course analysis carried out for 4 weeks demonstrated that these properties remained stable upon the time in culture.^9^ In addition, the optical characterization UGRSKIN model showed that the epidermal layer was able to mature and differentiate being able to absorb most of the incoming UV light, as it is the case of the native skin.^10^ When grafted in vivo on immune‐deficient animal models, UGRSKIN demonstrated to be highly biocompatible and able to contribute to skin regeneration.^8^ One of our main findings was that the bioartificial skin substitute was able to remodel its structure and composition in a time‐dependent manner, with a progressive process of extracellular matrix (ECM) remodeling and biosynthesis of crucial fibrillar and non‐fibrillar components along with cell differentiation and maturation.^8^, ^9^, ^12^ These positive results allowed us to fulfill the requirements of the Spanish Medicines Agency (AEMPS) for advanced therapy medicinal products (ATMP), thus allowing us to transfer this skin substitute to the clinical setting as compassionate use.^13^ Preliminary results of the clinical use of the UGRSKIN model in 12 severely burnt patients showed a 75% survival rate. Of the 12 patients, 9 showed a significant improvement and recovery, whereas three patients died mostly due to infections and systemic complications.^13^

Although results were encouraging, a comprehensive structural analysis of the histological and biochemical processes involved in tissue regeneration using the UGRSKIN model in humans is still in need. This analysis could provide valuable information regarding tissue biointegration and biofunctionality, and would contribute to a better characterization of the therapeutic effects of this type of ATMP according to the requirements established by the European Medicines Agency.^14^

In the present work, we have carried out a comprehensive structural and ultrastructural study of the UGRSKIN model grafted as ATMP in severely burnt patients after 1, 2, and 3 months of follow‐up in order to assess the biocompatibility of this model in humans and to determine the histological factors associated to the therapeutic effect of this bioartificial skin.

## 2 MATERIALS AND METHODS

### 2.1 Patients samples

The UGRSKIN model of bioartificial human skin was generated using autologous dermal fibroblasts, epidermal keratinocytes and fibrin‐agarose biomaterials as previously described.^8^, ^13^, ^15^ Cultured cells were first obtained from human skin biopsies obtained from patients treated at the burn unit of the University Hospital Virgen del Rocio in Seville (8 male and 4 female patients) and processed at the Cell Production and Tissue Engineering Unit of University Hospital Virgen de las Nieves in Granada, in a GMP facility of the Andalusian Network for the design and translation of Advanced Therapies (And&tAT). Dermal fibroblasts and keratinocytes were isolated using enzymatic digestion and cultured in specific culture media.^8^, ^15^ Once primary cell cultures were stablished, we generated a dermal substitute by combining cultured human fibroblasts with fibrin‐agarose hydrogels. Human plasma obtained from healthy blood donors was used as a source of fibrin. For 1 mL of dermal substitute, we mixed 760 μL of human plasma with 75 μL of DMEM containing 10,000 cultured dermal fibroblasts and 15 μL of tranexamic acid (Amchafibrin, Fides‐Ecofarma, Valencia, Spain). A total of 50 μL of a 2% melted solution of type VII agarose were added, followed by 100 μL of 1% CaCl_2_ (Merck, Darmstadt, Germany), and the mixture was immediately mixed and aliquoted in culture plates. After 24 h in an incubator at 37°C, 500,000 cultured keratinocytes were seeded on top of the stromal substitute. Finally, the UGRSKIN model was subjected to plastic compression nanostructuration and delivered to the burn unit as an ATMP for autologous use. All this biofabrication procedure as well as the clinical use in each patient were authorized by the Spanish Medicines Agency (AEMPS). Twelve severely burnt patients treated with the UGRSKIN model were included in this study (*n* = 12). The clinical characteristics of these patients were previously described.^13^ After 30, 60, and 90 days of follow‐up, a full‐thickness biopsy was obtained from the grafted area of each patient using a skin biopsy punch.

The study was conducted according to the guidelines of the Declaration of Helsinki and approved by the Institutional Ethics Committee of the Province of Granada (*Comité Ético de Investigación, CEIM/CEI protocol number S1900527*), and all skin donors provided informed consent for their participation in the study. All patients were treated with the approval of the Spanish Medicines Agency (AEMPS) using the hospital exemption and compassionate use for advanced therapy medicinal products (ATMP), as previously described.^13^

### 2.2 Histological, histochemical, and immunohistochemical analyses

Five types of samples were analyzed in the present work: ex vivo samples (UGRSKIN model of skin before clinical application), biopsies of patients treated with the UGRSKIN model after 30, 60, and 90 days of follow‐up and normal skin control samples. In each case, histological analysis was carried out using transmission electron microscopy (TEM) and light microscopy analysis. For TEM, samples were fixed in 2.5% buffered glutaraldehyde, postfixed in 1% osmium tetroxide, and dehydrated in graded acetone series. Then samples were embedded in Spurr's resin and stained with aqueous uranyl‐acetate and lead citrate. Ultrathin sections were examined using a EM902 microscope (Carl Zeiss Medi‐tec, Oberkochen, Germany). For light microscopy, samples fixed in 10% buffered formalin, embedded in paraffin, and sectioned in 5‐μm‐thick sections, which were stained with hematoxylin and eosin and histologically analyzed using a Nikon Eclipse 90i light microscope.

To analyze specific components of the human skin dermis ECM, the following histochemical methods were performed: to identify mature collagen fibers, the picrosirius red method was used and samples were observed using a light microscope. To assess the presence of mature, properly oriented collagen bundles, the same samples were analyzed using polarized light microscopy. Elastic fibers were stained by Verhoeff histochemistry, whereas proteoglycans were stained with alcian blue. In addition, Schiff periodic acid staining (PAS) methods were used to identity the basement membranes glycosaminoglycans. All these methods were used using routine protocols described elsewhere.^16^, ^17^, ^18^

Other relevant components of the human epidermis and dermis were identified using specific immunohistochemical procedures, including epithelial cytokeratins (CK5, CK8, and CK10), cell‐cell junctions markers (plakoglobin and claudin), epithelial differentiation proteins (filaggrin and involucrin), specific epithelial cells (melanocytes and Langerhans cells), components of the ECM (type‐V collagen and decorin), blood vessels (positive for CD31 and SMA) and lymphatic vessels (positive for D240), as well as proliferating cells (positive for Ki67). In brief, tissue sections were deparaffinized and rehydrated. Heat‐induced epitope retrieval was performed using citrate buffer pH 6.0 at 95°C and samples were prehybridized with normal horse serum (Vector Laboratories, Burlingame, CA) for 1 h and incubated with primary antibodies overnight (see Supplementary Table S1 for references and concentrations). After rinsing, samples were hybridized with peroxidase‐conjugated secondary antibodies for 1 h at room temperature and revealed with diaminobenzidine‐DAB‐(Vector Laboratories). Finally, tissue sections were counterstained with Harris hematoxylin for 5 s, rinsed, dehydrated, and mounted. For claudin and plakoglobin samples were analyzed by immunofluorescence. In both cases, tissue sections were processed as described above, but secondary antibodies conjugated with Cy3 or FITC (Sigma‐Aldrich) were used at 1:500 dilution, and samples were counterstained with DAPI mounting medium (Vector Laboratories, Burlingame, CA, United States).

### 2.3 Quantitative analyses

All tissue sections subjected to histochemical and immunohistochemical analysis were analyzed using a Nikon Eclipse 90i light microscope (Nikon Corp., Tokyo, Japan). In each case, images were obtained from each sample subjected to the same technique using exactly the same parameters and configuration (exposure, intensity, background, white balance, etc.). Then, images were analyzed to quantify the intensity signal using a quantitative approach method as previously reported for the human skin.^18^

On the one hand, the number of blood and lymphatic vessels found per mm^2^ of superficial or profound dermis was independently quantified by three independent histologists, and averages were calculated. In the case of Ki67, the number of cells showing positive signal was quantified per mm of the basal layer of the epidermis and expressed as number of cells per mm of epithelium (CPM), since proliferating cells reside at the basal stratum of the epidermis. The same method was used to determine the number of cells showing positive staining signal for melanocytes and Langerhans cell markers.

Furthermore, the results of the histochemical and immunohistochemical analyses were quantified using an automatic method based on the ImageJ software (National Institutes of Health, USA). For picrosirius red with non‐polarized light, alcian blue, and PAS histochemistry, and for the immunohistochemical expression of epidermal markers (cytokeratins, cell‐cell junctions, and differentiation markers), type‐V collagen and decorin, average sample intensity was calculated by randomly selecting 10 points in each type of sample (*n* = 10), as previously described.^19^, ^20^ For polarized light picrosirius red and Verhoeff, we calculated the area of the dermis that was occupied by the stained fibers by using the area fraction option of the software. In the case of the dermal markers, the superficial and the profound dermis were analyzed separately.

### 2.4 Statistical analysis

For each sample and for each analysis method, results were expressed as averages and standard errors of the mean, and as medians and quartile ranges (corresponding to the first and third quartiles). Results of the quantitative analyses were compared with control native human skin and with ex vivo samples. First, each distribution was evaluated using the Shapiro–Wilk test to determine the normality of each distribution. Results of this analysis showed that the distributions were not normal and, therefore, did not fulfill the criteria for parametric analysis. Therefore, comparison of the results obtained for each type of sample were compared with controls and with ex vivo samples using the Mann–Whitney *U* test, which is designed for the comparison of two non‐parametric distributions. These analyses were performed using the *Real Statistics* software and *p* values below 0.05 were considered statistically significant for the double‐tailed tests.

## 3 RESULTS

### 3.1 Characterization of the epidermal layer of UGRSKIN grafted in patients

#### 3.1.1 Histological characterization of the epidermis using light and electron microscopy

**FIGURE 1:**
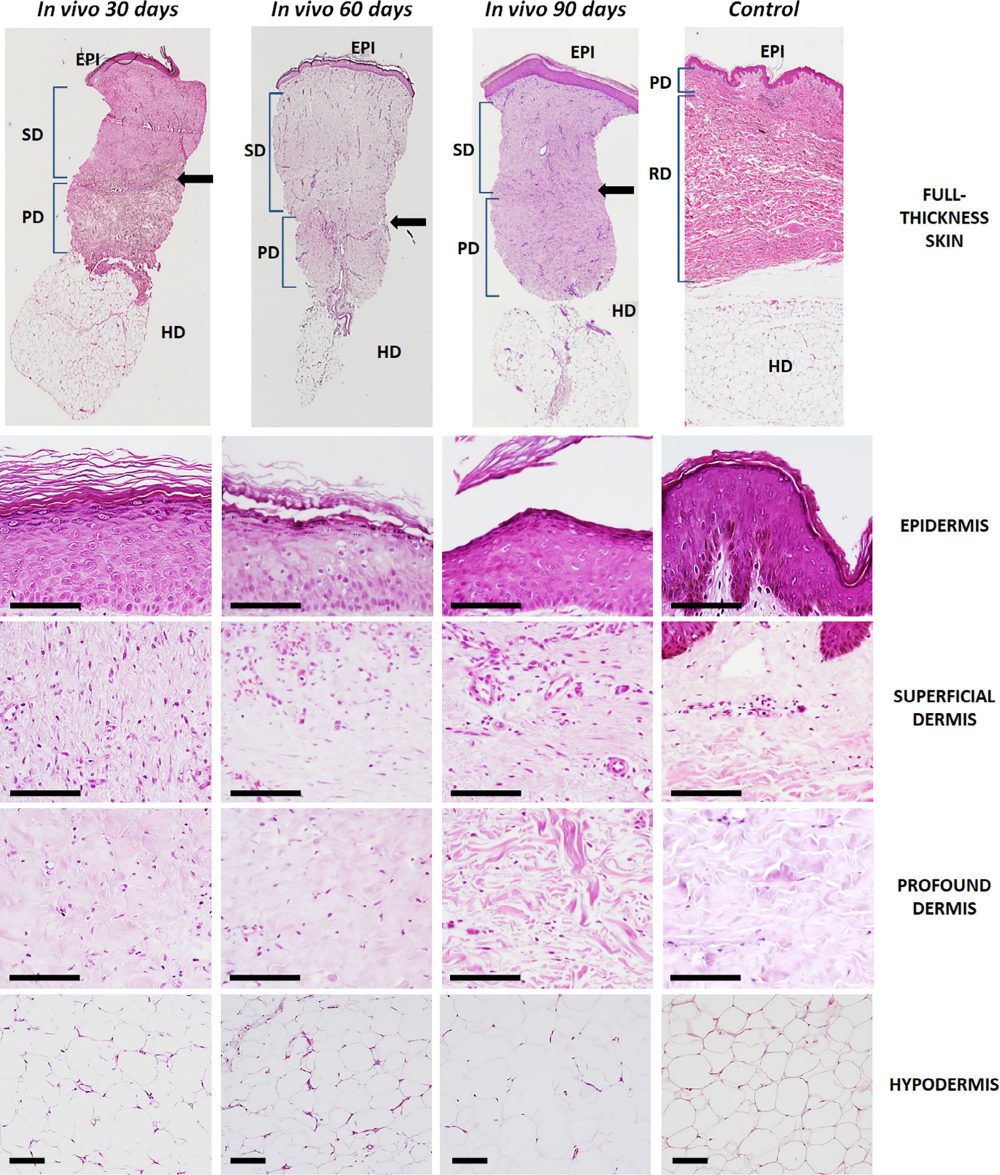
Analysis of UGRSKIN grafted in patients and control native skin using HE staining. The top panel shows a low magnification image of the epidermis, dermis and hypodermis layers of the skin (full‐thickness), whereas each layer is shown at higher magnification in the lower panels. EPI, epidermis; HD, hypodermis; PD, profound dermis; SD, superficial dermis. The interface between the superficial and profound dermis is labeled with arrows. Scale bars: 100 μm.

Analysis of the epidermal layer of UGRSKIN grafted in patients for 30, 60, and 90 days and stained with HE revealed that this layer was highly differentiated and properly integrated at all study times. As shown in Figure 1, the grafted skin epidermis consisted of four cell strata that were analogue to control native human skin (basal, spinosum, granulosum and corneum strata). However, the skin was devoid of the typical epithelial rete ridges and dermal papillae found in control skin.

**FIGURE 2:**
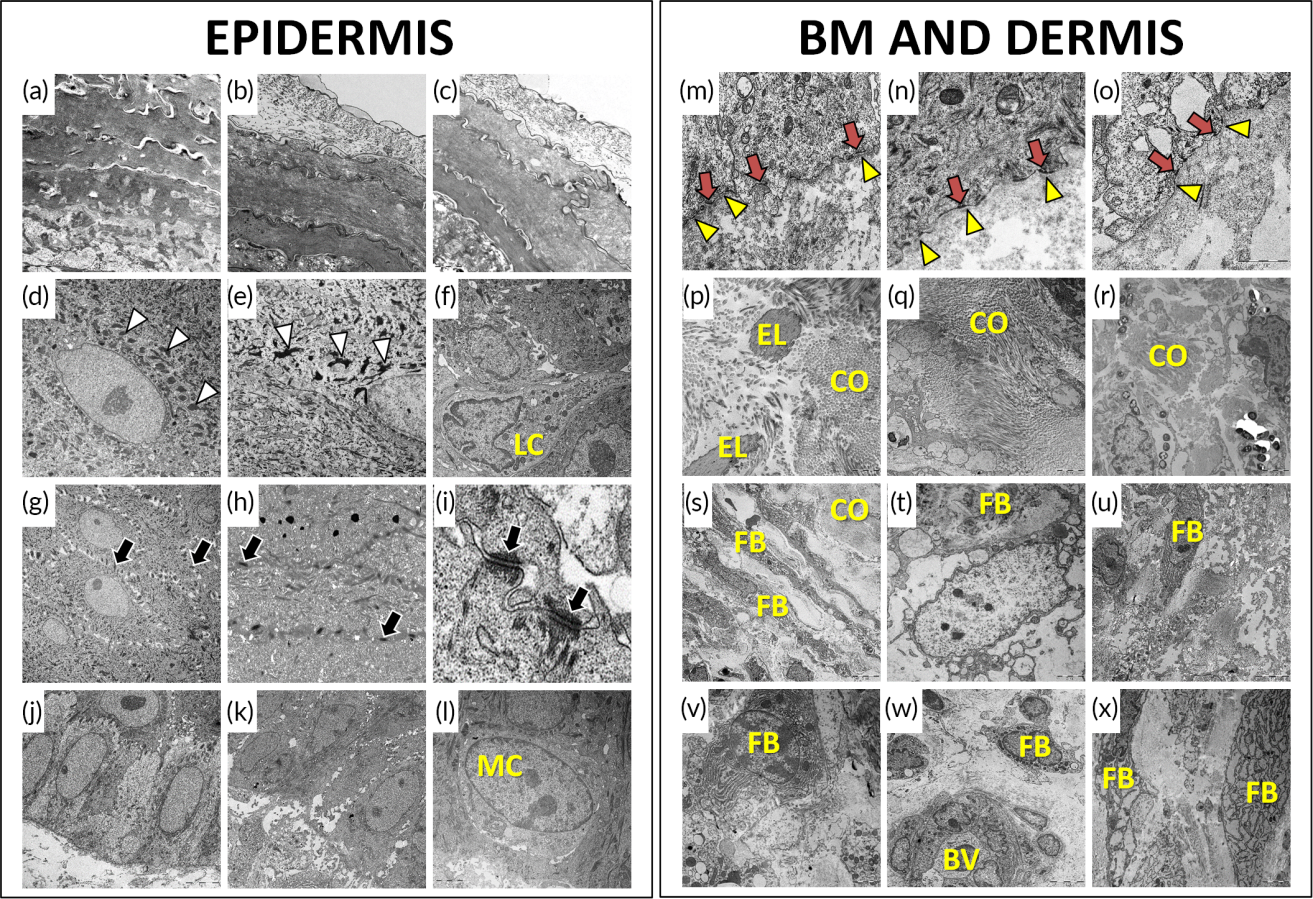
TEM analysis of UGRSKIN grafted in patients. The left panel corresponds to the analysis of the epidermal layer, whereas the basement layer and dermis are shown at the right. (a–c) corneum stratum of UGRSKIN after 30, 60, and 90 days of follow‐up; (d–f) granulosum stratum; (g, h, i) spinosum stratum; (j–l) basal stratum; (m–o) basement membrane; (p–r): fibers of the dermal layer; (s–x) cells at the dermal layer. BV, blood vessel; CO, collagen fibers; EL, elastin fibers; FB, fibroblasts; LC, Langerhans cell; MC, melanocyte cell. White arrowheads: keratohyaline granules; black arrows: desmosome cell‐cell junctions; yellow arrowheads: basement membrane; red arrows: hemidesmosomes.

At the ultrastructural level (Figure 2), analysis of samples using TEM confirmed that the epidermal layer was highly differentiated at all study times. First, we found that the superficial stratum of all samples consisted of several layers of terminally differentiated keratinocytes (skin corneocytes). Then, we found that the granulosum stratum was composed by epithelial keratinocytes containing abundant keratohyalin electron‐dense granules. At the spinosum stratum, cells showed numerous intercellular junctions, especially, desmosomes. Finally, the basal stratum consisted of elongated keratinocytes whose shape was compatible with the basal cells of the human epithelium. Interestingly, isolated Langerhans cells and melanocytes were detected within the epidermal layer. No differences were found among samples corresponding to 30, 60, and 90 days of follow‐up.

#### 3.1.2 Immunohistochemical and immunofluorescence characterization of the skin epidermis

**FIGURE 3:**
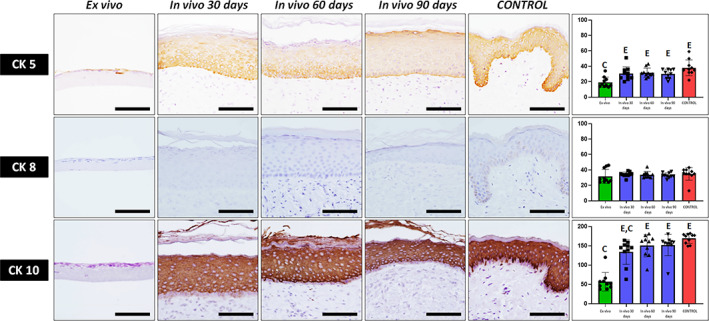
Immunohistochemical analysis of cytokeratins 5, 8, and 10 expression in the epidermal layer of UGRSKIN kept ex vivo, UGRSKIN grafted in patients for 30, 60, and 90 days and control human native skin. Scale bars: 100 μm. Histograms to the right show the average and standard deviation results of the staining signal quantification for each marker expressed as intensity units (I.U.). Significant differences with control native skin are labeled with C, whereas significant differences with samples kept ex vivo are labeled with E. Statistical comparisons were carried out with 10 samples per experimental group.

Analysis of epithelial markers at the epidermal layer was carried out by immunofluorescence and immunohistochemistry. First, we assessed the expression of three epithelial cytokeratins (Figure 3 and Tables 1 and 2). For CK8, our results revealed that all samples were negative, and no differences with the control native skin were detected. For CK5, we found that that the lowest expression corresponded to the UGRSKIN samples kept in culture, with differences with control native skin being statistically significant. However, differences between samples grafted in patients and controls were nonsignificant. When CK10 was analyzed, we found very low staining signal in samples kept ex vivo, whereas controls and in vivo samples showed very strong signal. Differences with controls were statistically significant for samples kept ex vivo and 30‐days in vivo skin tissues (Figure 3 and Tables 1 and 2).

**TABLE 1:** Histochemical and immunohistochemical analysis of the main epithelial markers and histochemical staining of the basement membrane using PAS in control native skin and UGRSKIN kept ex vivo and grafted in patients for 30, 60, and 90 days.

Marker	Layer	UGRSKIN ex vivo	UGRSKIN 30 days	UGRSKIN 60 days	UGRSKIN 90 days	Control
CK5	Epidermis	19.2 ± 2.1	30.4 ± 2.8	31.6 ± 1.9	30.1 ± 1.9	38.0 ± 3.2
		16.0 [14.3–22.5]*	29.0 [25.0–34.0]	30.5 [29.0–31.8]	31.0 [25.0–35.8]	35.5 [32.8–40.3]
CK8	Epidermis	31.7 ± 2.9	34.2 ± 1.0	33.7 ± 1.4	33.0 ± 1.0	34.8 ± 2.6
		27.5 [25.3–39.5]	34.0 [33.3–35.0]	32.5 [31.0–35.5]	32.5 [32.0–35.3]	35.5 [35.0–39.5]
CK10	Epidermis	56.8 ± 7.6	134.1 ± 10.2	150.2 ± 9.1	152.0 ± 8.7	169.1 ± 4.0
		51.5 [43.8–57.8]*	145.0 [136.0–151.3]	161.5 [132.8–167.5]	158.0 [155.0–160.8]	176.0 [159.0–178.5]
Claudin	Epidermis	24.6 ± 7.6	98.4 ± 14.3	107.2 ± 11.0	105.6 ± 22.4	119.1 ± 10.4
		14.5 [4.0–44.8]*	103.5 [62.0–117.3]	102.5 [95.3–114.0]	89.5 [70.0–143.0]	125.5 [93.8–146.5]
Plakoglobin	Epidermis	50.2 ± 11.6	90.3 ± 10.2	93.2 ± 7.6	98.5 ± 3.8	87.5 ± 4.8
		59.0 [21.8–79.3]*	96.5 [71.0–116.8]	96.0 [90.3–108.0]	101.0 [94.0–105.8]	83.0 [78.8–87.8]
Filaggrin	Epidermis	65.3 ± 7.6	175.8 ± 7.4	179.2 ± 14.8	186.4 ± 6.1	170.7 ± 5.4
		57.0 [55.5–66.3]*	170.5 [161.3–194.8]	189.5 [154.5–211.3]	193.5 [173.5–197.8]	170.5 [161.0–180.8]
Involucrin	Epidermis	34.1 ± 4.5	66.9 ± 3.6	85.4 ± 5.1	89.0 ± 3.5	96.1 ± 5.1
		32.5 [28.3–38.5]*	67.0 [57.8–78.0]*	83.5 [71.0–96.3]	87.0 [79.5–97.3]	98.0 [92.8–106.0]
Melan‐A	Epidermis	0.0 ± 0.0	0.2 ± 0.1	17.8 ± 0.7	14.4 ± 1.4	28.6 ± 1.4
		0.0 [0.0–0.0]*	0.0 [0.0–0.0]*	18.5 [15.3–19.8]*	12.0 [11.0–18.8]*	27.5 [25.3–32.8]
CD1a	Epidermis	0.0 ± 0.0	5.8 ± 0.2	19.0 ± 1.1	23.2 ± 1.0	61.2 ± 1.7
		0.0 [0.0–0.0]*	6.0 [5.0–6.0]*	19.5 [16.5–20.0]*	22.5 [21.0–26.5]*	61.5 [58.0–65.8]
Ki67	Epidermis	44.2 ± 2.6	44.6 ± 2.2	51.4 ± 0.8	64.6 ± 1.5	60.0 ± 2.6
		41.5 [38.5–50.8]*	42.5 [40.5–48.0]*	51.0 [50.0–52.8]	62.5 [61.0–68.8]	60.0 [56.0–64.8]
PAS	Basement membrane	10.3 ± 4.3	53.4 ± 4.6	60.8 ± 6.0	58.8 ± 12.5	63.7 ± 5.2
		3.5 [2.0–11.8]*	54.5 [40.8–63.8]	60.0 [48.8–77.0]	48.5 [40.3–52.5]	64.0 [53.0–72.3]

*Note*: Results correspond to the quantitative analysis of staining intensity, except for Melan‐A, CD1a and Ki67, corresponding to the number of positive cells found per millimeter of epidermis. Results are shown as average ± standard error of the mean followed by the median and quartile range [first and third quartiles].

* *p* values below 0.05 as compared to control native skin are labeled with asterisks.

**TABLE 2:** Statistical analysis of the quantitative results obtained by histochemistry and immunohistochemistry.

Marker	Layer	Control vs. UGRSKIN ex vivo	Control vs. UGRSKIN 30 days	Control vs. UGRSKIN 60 days	Control vs. UGRSKIN 90 days	UGRSKIN ex vivo vs. UGRSKIN 30 days	UGRSKIN ex vivo vs. UGRSKIN 60 days	UGRSKIN ex vivo vs. UGRSKIN 90 days	UGRSKIN 30 days vs. UGRSKIN 60 days	UGRSKIN 30 days vs. UGRSKIN 90 days	UGRSKIN 60 days vs. UGRSKIN 90 days
CK5	Epidermis	0.0002*	0.0524	0.0524	0.0753	0.0039*	0.0011*	0.0021*	0.5787	0.7394	0.8534
CK8	Epidermis	0.315	0.1431	0.1431	0.0753	0.1903	0.123	0.1903	0.4813	0.2176	0.9118
CK10	Epidermis	<0.0001*	0.0007*	0.123	0.123	0.0001*	<0.0001*	<0.0001*	0.2176	0.0288*	0.6305
Claudin	Epidermis	<0.0001*	0.1431	0.393	0.4359	0.0003*	0.0001*	0.0015*	0.7394	0.8534	0.7394
Plakoglobin	Epidermis	0.0288*	0.5288	0.2176	0.1051	0.0185*	0.0052*	0.0005*	0.9705	0.8534	0.8534
Filaggrin	Epidermis	<0.0001*	0.6842	0.315	0.063	<0.0001*	<0.0001*	<0.0001*	0.6305	0.3527	0.9705
Involucrin	Epidermis	<0.0001*	0.0005*	0.1431	0.123	0.0002*	<0.0001*	<0.0001*	0.0232*	0.0007*	0.4359
Melan‐A	Epidermis	<0.0001*	<0.0001*	<0.0001*	<0.0001*	0.4813	<0.0001*	<0.0001*	<0.0001*	<0.0001*	0.1051
CD1a	Epidermis	<0.0001*	<0.0001*	<0.0001*	<0.0001*	<0.0001*	<0.0001*	<0.0001*	<0.0001*	<0.0001*	0.0115*
Ki67	Epidermis	0.0007*	0.0005*	0.0147*	0.2475	0.6842	0.0753	<0.0001*	0.0232*	<0.0001*	<0.0001*
PAS	Basement membrane	<0.0001*	0.1903	0.6305	0.123	<0.0001*	<0.0001*	0.0001*	0.4359	0.5787	0.2475
Picrosirius red	Superficial dermis	<0.0001*	0.0147*	0.0039*	0.2475	0.0052*	0.0005*	<0.0001*	0.123	0.063	0.1903
	Profound dermis	<0.0001*	0.7394	0.9118	0.393	<0.0001*	<0.0001*	<0.0001*	0.9118	0.2475	0.6842
Polarized picrosirius red	Superficial dermis	0.0079*	0.0079*	0.0079*	0.9999	0.0079*	0.0079*	0.0079*	0.0079*	0.0079*	0.0952
	Profound dermis	0.0079*	0.0079*	0.6905	0.5476	0.0079*	0.0079*	0.0079*	0.0079*	0.0079*	0.0317*
Type‐V collagen	Superficial dermis	<0.0001*	0.0355*	0.0147*	0.0089*	<0.0001*	<0.0001*	<0.0001*	0.0185*	0.0005*	<0.0001*
	Profound dermis	<0.0001*	0.7959	0.0005*	0.0001*	<0.0001*	<0.0001*	<0.0001*	0.0355*	0.0039*	0.393
Verhoeff	Superficial dermis	0.0079*	0.0079*	0.3095	0.2222	0.1508	0.0079*	0.0159*	0.0079*	0.1508	0.5476
	Profound dermis	0.0079*	0.0079*	0.0079*	0.0079*	0.0159*	0.0079*	0.0079*	0.0079*	0.0079*	0.0159*
Alcian blue	Superficial dermis	0.0232*	0.0185*	0.063	0.2799	0.0288*	0.8534	0.0433*	0.0068*	0.0892	0.7394
	Profound dermis	0.063	0.1431	0.8534	0.9118	0.0115*	0.0288*	0.0753	0.2475	0.1051	0.8534
Decorin	Superficial dermis	0.0089*	0.0185*	0.9705	0.5787	0.5787	0.0115*	0.0007*	0.0007*	0.3527	0.5288
	Profound dermis	0.0005*	0.8534	0.5288	0.9118	0.0002*	<0.0001*	0.0003*	0.7394	0.4359	0.5787
CD31	Superficial dermis	<0.0001*	<0.0001*	<0.0001*	<0.0001*	<0.0001*	<0.0001*	<0.0001*	<0.0001*	<0.0001*	<0.0001*
	Profound dermis	0.0002*	<0.0001*	<0.0001*	<0.0001*	<0.0001*	<0.0001*	<0.0001*	0.2799	0.9705	0.2475
SMA	Superficial dermis	<0.0001*	<0.0001*	<0.0001*	<0.0001*	<0.0001*	<0.0001*	<0.0001*	<0.0001*	<0.0001*	<0.0001*
	Profound dermis	0.0002*	<0.0001*	<0.0001*	0.0001*	<0.0001*	<0.0001*	<0.0001*	0.0039*	<0.0001*	0.0002*
D240	Superficial dermis	<0.0001*	<0.0001*	0.8534	<0.0001*	0.063	0.0002*	<0.0001*	0.4359	0.0115*	<0.0001*
	Profound dermis	0.0002*	0.5288	0.0892	0.7394	0.063	0.0002*	0.0002*	0.0433*	0.2799	0.1903

*Note*: Values correspond to statistical *p* values for the comparison of two specific groups using the Mann–Whitney *U* statistical test.

* *p* values below 0.05 are labeled with asterisks.

**FIGURE 4:**
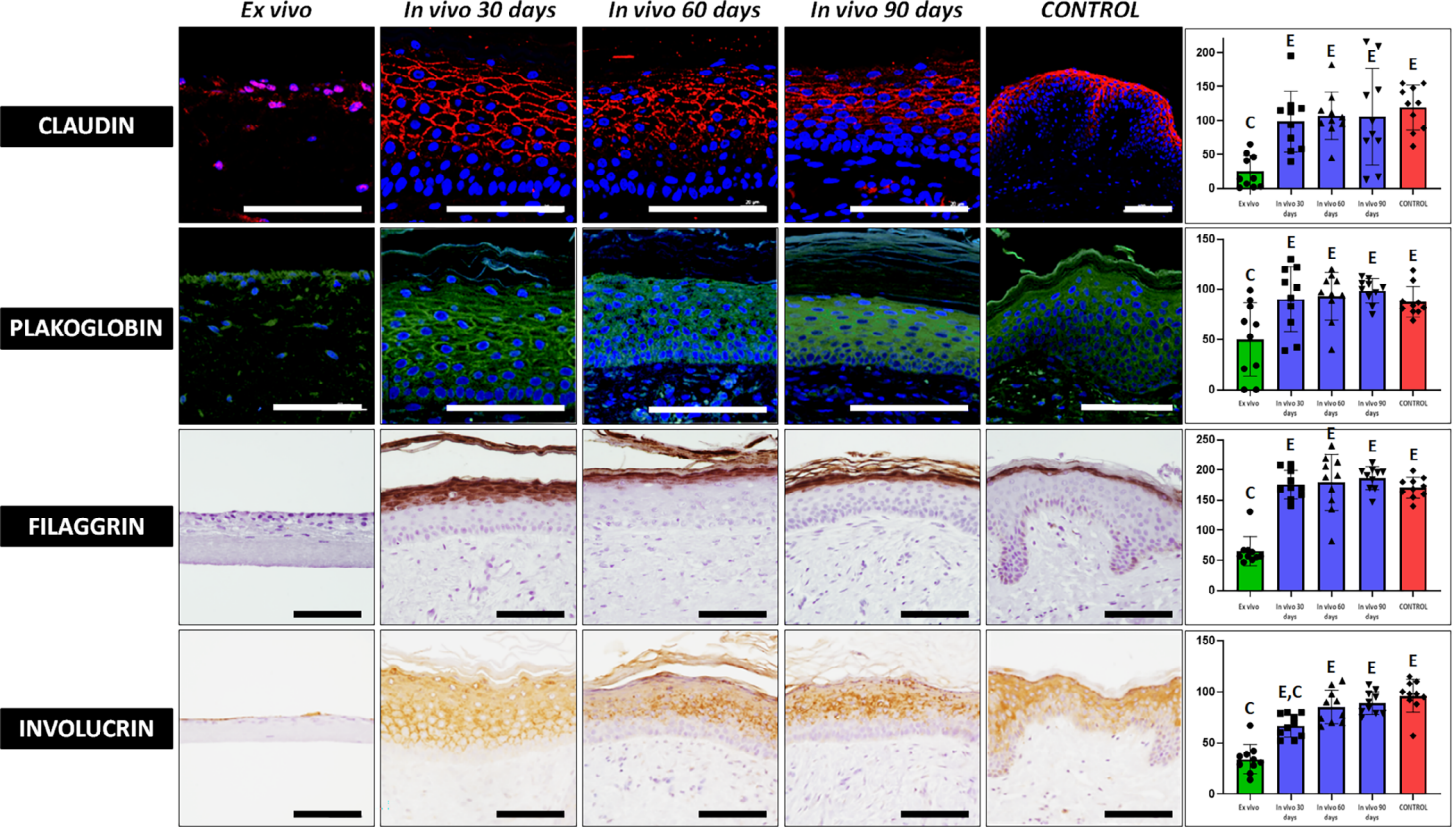
Immunofluorescence and histochemical analysis of claudin, plakoglobin, filaggrin and involucrin expression in the epidermal layer of UGRSKIN kept ex vivo, UGRSKIN grafted in patients for 30, 60, and 90 days and control human native skin. Scale bars: 100 μm. Histograms to the right show the average and standard deviation results of the staining signal quantification for each marker expressed as intensity units (I.U.). Significant differences with control native skin are labeled with C, whereas significant differences with samples kept ex vivo are labeled with E. Statistical comparisons were carried out with 10 samples per experimental group.

Then, we evaluated expression of two types of cell‐cell junctions proteins playing a key role in maintaining the epithelial barrier function in each type of sample. As shown in Figure 4 and Tables 1 and 2, we found strong positive immunofluorescence expression of claudin and plakoglobin proteins in grafted skin from day 30 onward, with values comparable to control native skin, although expression was very low in ex vivo samples, which were significantly lower than control skin.

In addition, we found that the bioartificial human skin keratinocytes expressed filaggrin and involucrin, two proteins related to terminal differentiation of the human skin epidermis. As shown in Figure 4 and Tables 1 and 2, suprabasal strata showed strong positive immunohistochemical signal for these two proteins, as it was the case of control skin. In contrast, expression was slight or negative in ex vivo samples. Differences with controls were significant for ex vivo samples and for 30‐days in vivo skin in the case of involucrin.

**FIGURE 5:**
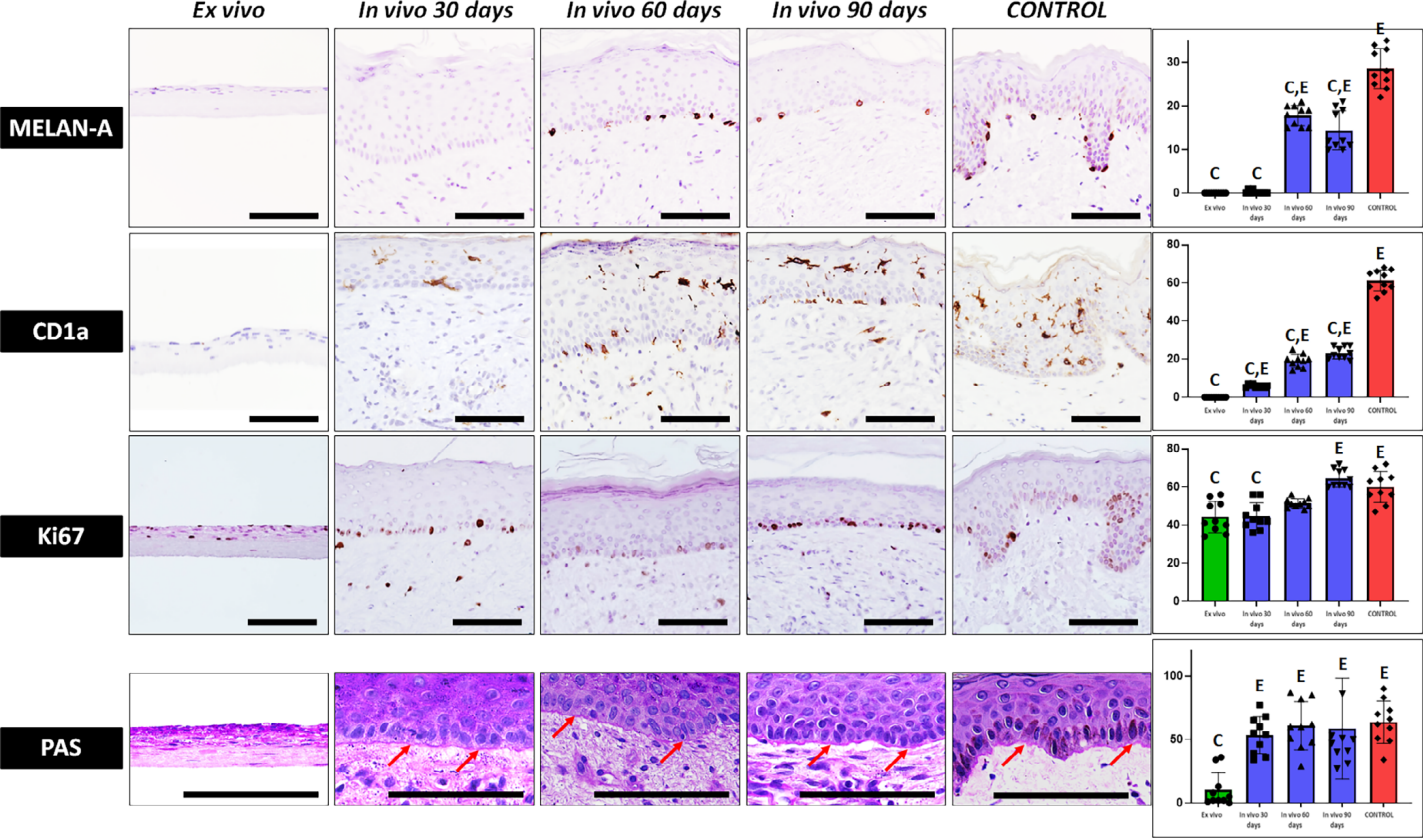
Identification of epidermal melanocytes, Langerhans cells and proliferating cells using MELAN‐A, CD1a and Ki67 immunohistochemistry, respectively and histochemical staining of the basement membrane using PAS histochemical method. Illustrative positive signal for the PAS staining is labeled with arrows. Scale bars: 100 μm. Histograms to the right show the average and standard deviation results of the quantitative analysis of cells showing positive expression of MELAN‐A, CD1a and Ki67 (expressed as number of positive cells per mm of epithelium, CPM) and PAS staining signal quantification at the basement membrane (expressed as intensity units, I.U.). Significant differences with control native skin are labeled with C, whereas significant differences with samples kept ex vivo are labeled with E. Statistical comparisons were carried out with 10 samples per experimental group.

Furthermore, we quantified the number of melanocytes and Langerhans cells in the epithelium of UGRSKIN after identifying each cell type by immunohistochemistry (Figure 5 and Tables 1 and 2). In this regard, we found that control skin had an average and standard error of 28.6 ± 1.4 melanocytes per mm of epithelium (CPM), whereas ex vivo samples were devoid of these cells (0.0 ± 0.0 CPM) and skin grafted in patients showed a progressively increasing number of melanocytes (0.2 ± 0.1 CPM at day 30, 17.8 ± 0.7 at day 60 and 14.4 ± 1.4 at day 90 of follow‐up). A very similar situation was found for Langerhans cells, with controls showing 61.2 ± 1.7 CPM of epithelium and samples grafted in patients showing 5.8 ± 0.2, 19 ± 1.1 and 23.2 ± 1.0 CPM after 30, 60, and 90 days, respectively, whereas ex vivo samples were negative (0.0 ± 0.0 CPM). Differences with control native skin were statistically significant for both the melanocytes and Langerhans cells number (*p* < 0.05) (Table 2).

Finally, analysis of proliferating cells showing positive Ki67 signal (Figure 5 and Tables 1 and 2) revealed the presence of 60.0 ± 2.6 proliferating CPM of epithelium of native control skin. However, the number of proliferating cells was significantly lower (*p* < 0.05) in ex vivo samples (44.2 ± 2.6 CPM) and in skin grafted in patients for 30 days (44.6 ± 2.2 CPM), although no statistical differences with controls were found at 60 and 90 days (51.4 ± 0.8 and 64.6 ± 1.5 CPM, respectively).

### 3.2 Characterization of the human skin basement membrane by histochemistry and electron microscopy

Histochemical staining of UGRSKIN using the PAS method showed that all skin biopsies obtained from patients grafted with this skin model showed positive staining at the basement membrane, and the staining intensity was similar to control native human skin, whereas ex vivo samples showed significantly lower PAS staining intensity (Figure 5 and Tables 1 and 2). In addition, we confirmed the presence of a well‐differentiated basement membrane at the ultrastructural level using TEM (Figure 2). In this regard, we found that skin grafted in patients for 30, 60, and 90 days showed a well‐defined basement membrane at the dermo‐epidermal junction, which was associated to numerous hemidesmosomes in basal keratinocytes (Figure 2).

### 3.3 Characterization of the dermal layer of UGRSKIN grafted in patients

#### 3.3.1 Histological characterization of the dermal layer using light and electron microscopy

When the dermis of the grafted skin was evaluated using HE staining (Figure 1), we found that this layer consisted of a dense fibrillar structure in which abundant stromal cells and blood vessels were immersed. As expected, control native skin showed a superficial papillary dermis with numerous cells and capillaries, and a profound reticular dermis rich in collagen fibers. However, the dermo‐epidermal junction of UGRSKIN grafted in patients was flat, and a clear distinction between papillary and reticular dermis was not found. Interestingly, all in vivo samples showed an interface separating a superficial dermis from a profound dermis, especially in the case of the biopsies corresponding to 30 days of follow‐up, which probably corresponds to the limit between the grafted bioartificial skin and the remaining native connective tissue of the patient. This interface tended to progressively disappear with time and was almost absent in 90‐days biopsies. Analysis of the superficial dermis of in vivo samples showed abundant cells and vessels, as it was the case of the controls. However, the profound dermis was richer in collagen fibers and contained less stromal cells, especially in biopsies taken after 90 days of follow‐up, whilst 30‐days biopsies showed numerous cells immersed in the dermal extracellular matrix. No differences were found between the native skin and the in vivo samples for the hypodermal layer of the human skin (Figure 1).

Ultrastructural evaluation of the dermis of grafted UGRSKIN showed that the dermis was well‐differentiated in all in vivo samples (Figure 2). All samples contained an abundant extracellular matrix with capillary blood vessels and numerous collagen and elastic fibers. Dermal fibroblasts containing abundant organelles such as ribosomes and endoplasmic reticulum were found, suggesting that these cells were metabolically active at all study times. No differences were found among the different in vivo samples at the ultrastructural level.

#### 3.3.2 Histochemical and immunohistochemical characterization of the dermal layer

Fibrillar and non‐fibrillar components of the human skin dermis extracellular matrix were assessed by histochemistry and immunohistochemistry.

**FIGURE 6:**
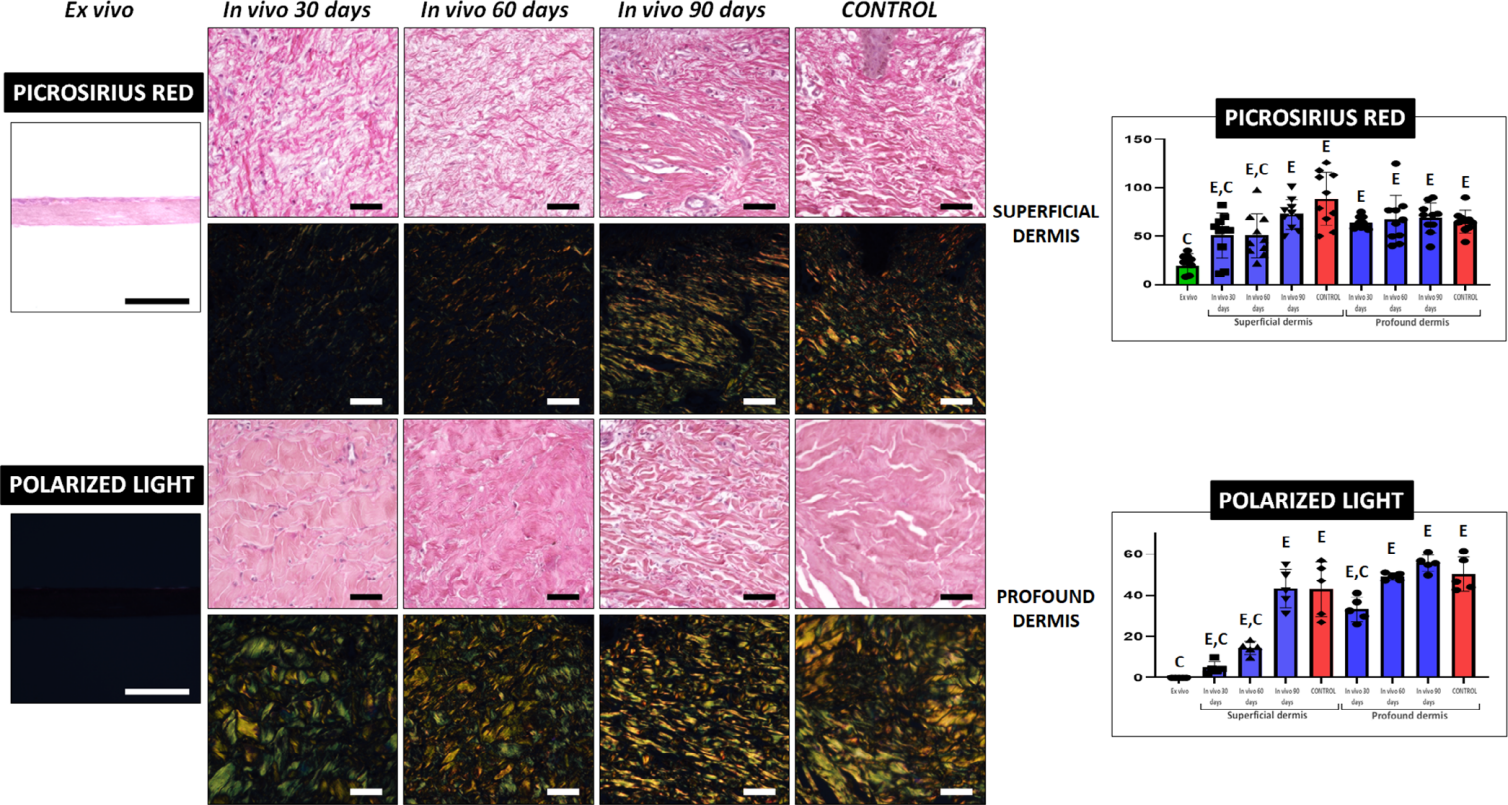
Analysis of mature collagen fibers in control skin and UGRSKIN using picrosirius red histochemical method. In each type of sample, images were taken with non‐polarized and polarized light at the superficial and profound dermis. Scale bars: 100 μm. Histograms to the right show the average and standard deviation results of the staining signal quantification for each marker expressed as intensity units (I.U.). Significant differences with control native skin are labeled with C, whereas significant differences with samples kept ex vivo are labeled with E. Statistical comparisons were carried out with 10 samples per experimental group.

First, the presence of collagen fibers was assessed by picrosirius red histochemistry (Figure 6 and Tables 2 and 3). Results showed that the signal intensity of UGRSKIN samples was significantly lower in ex vivo samples as compared to control skin. For the superficial dermis, intensity of biopsies at 30 and 60 days was significantly lower than controls (*p* < 0.05), but samples at 90 days were similar to control skin. Interestingly, differences with control skin were nonsignificant for the profound dermis. Then, we wanted to assess the presence of mature and properly oriented collagen bundles using polarized light microscopy (Figure 6 and Tables 2 and 3). For the superficial dermis, we found that the area occupied by mature collagen was significantly higher in control skin as compared to ex vivo samples and biopsies at days 30 and 60. For the profound dermis, biopsies taken after 30 days had significantly less area occupied by mature collagen than controls, whereas in vivo samples at 60 and 90 days were similar to native skin.

**TABLE 3:** Histochemical and immunohistochemical analysis of the main dermal markers analyzed in this work in control native skin and UGRSKIN kept ex vivo and grafted in patients for 30, 60, and 90 days.

Marker	Layer	UGRSKIN ex vivo	UGRSKIN 30 days	UGRSKIN 60 days	UGRSKIN 90 days	Control
Picrosirius red	Superficial dermis	19.6 ± 3.8 22.9 [11.7–28.2]*	50.7 ± 7.4	50.5 ± 7.2	72.3 ± 4.9	88.7 ± 8.7
			56.4 [42.9–63.7]*	44.4 [35.7–64.7]*	71.9 [61.7–79.2]	86.9 [69.4–112.4]
	Profound dermis		63.8 ± 1.9	67.2 ± 7.9	68.9 ± 4.9	65.0 ± 3.7
			62.9 [59.2–68.4]	64.9 [50.2–76.9]	71.9 [61.9–76.7]	64.9 [60.2–68.4]
Polarized picrosirius red	Superficial dermis	0.0 ± 0.0 0.0 [0.0–0.0]*	4.8 ± 1.3	14.4 ± 1.4	43.2 ± 4.2	43.0 ± 6.0
			4.3 [3.4–4.5]*	14.9 [13.8–15.3]*	42.4 [38.2–49.8]	47.1 [31.0–53.1]
	Profound dermis		33.2 ± 2.6	49.1 ± 0.8	55.6 ± 1.8	50.3 ± 3.7
			32.5 [29.8–36.8]*	49.3 [47.4–50.4]	55.7 [54.9–56.8]	46.7 [44.0–57.0]
Type‐V collagen	Superficial dermis	23.9 ± 0.9 24.0 [22.0–24.0]*	92.9 ± 4.9	117.1 ± 8.0	126.6 ± 7.0	82.4 ± 12.9
			88.5 [83.5–103.5]*	119.0 [98.5–131.5]*	121.5 [116.8–145.8]*	67.0 [60.0–76.0]
	Profound dermis		71.7 ± 5.3	91.5 ± 5.3	95.2 ± 4.0	66.5 ± 5.0
			68.5 [59.3–84.8]	86.5 [84.3–95.5]*	95.0 [88.5–100.0]*	73.5 [60.5–76.3]
Verhoeff	Superficial dermis	0.3 ± 0.3 0.0 [0.0–0.0]*	0.4 ± 0.1	2.9 ± 0.6	2.6 ± 0.6	3.4 ± 0.1
			0.4 [0.3–0.5]*	2.3 [2.0–3.4]	1.8 [1.7–3.1]	3.5 [3.1–3.5]
	Profound dermis		1.8 ± 0.2	6.4 ± 0.7	4.7 ± 0.1	3.5 ± 0.3
			1.7 [1.7–1.9]	6.3 [5.4–6.3]	4.8 [4.4–4.9]	3.3 [2.7–4.2]
Alcian blue	Superficial dermis	31.3 ± 4.7 34.5 [26.3–39.3]	16.6 ± 2.7	38.6 ± 9.0	58.9 ± 8.5	72.0 ± 13.0
			16.0 [12.5–19.3]*	33.0 [25.3–49.8]	67.0 [32.8–82.8]	85.0 [61.5–98.5]
	Profound dermis		66.8 ± 11.3	51.6 ± 6.9	48.1 ± 8.2	49.3 ± 7.4
			68.5 [59.5–88.0]	47.5 [38.3–63.3]	47.0 [36.3–58.0]	48.0 [37.3–66.8]
Decorin	Superficial dermis	42.2 ± 5.4 41.0 [28.5–51.3]*	45.0 ± 6.7	77.8 ± 11.0	83.9 ± 7.9	80.9 ± 11.8
			50.0 [29.0–61.5]*	80.0 [54.0–95.5]	87.5 [67.5–96.5]	71.5 [57.0–104.3]
	Profound dermis		89.5 ± 7.6	93.8 ± 7.8	82.9 ± 5.8	87.5 ± 9.5
			96.0 [69.0–100.0]	85.0 [77.3–106.3]	85.5 [76.0–90.5]	77.0 [74.3–105.8]
CD31	Superficial dermis	0.0 ± 0.0 0.0 [0.0–0.0]*	104.0 ± 4.0	88.0 ± 7.3	111.0 ± 4.8	37.0 ± 1.9
			99.5 [95.3–113.0]*	90.0 [78.3–106.3]*	110.5 [101.3–123.8]*	37.0 [35.0–39.5]
	Profound dermis		30.0 ± 1.3	34.0 ± 2.8	30.0 ± 1.4	10.0 ± 2.2
			29.0 [27.0–34.0]*	37.5 [25.5–40.0]*	29.0 [26.3–34.5]*	10.5 [5.0–15.8]
SMA	Superficial dermis	0.0 ± 0.0 0.0 [0.0–0.0]*	105.0 ± 7.1	125.0 ± 13.0	128.0 ± 6.0	39.0 ± 2.2
			97.5 [90.3–109.0]*	111.0 [97.8–168.0]*	128.0 [122.0–139.8]*	39.5 [36.0–43.8]
	Profound dermis		44.0 ± 2.5	33.0 ± 2.0	19.0 ± 1.8	8.0 ± 1.6
			40.0 [38.5–51.5]*	31.0 [30.0–35.0]*	17.0 [15.0–19.8]*	9.5 [5.3–10.0]
D240	Superficial dermis	0.0 ± 0.0 0.0 [0.0–0.0]*	2.0 ± 0.8	10.0 ± 1.7	27.0 ± 1.8	11.0 ± 1.2
			0.5 [0.0–4.8]*	10.5 [9.3–14.8]	30.0 [21.3–30.8]*	10.5 [9.3–14.5]
	Profound dermis		4.0 ± 1.5	9.0 ± 1.9	6.0 ± 1.3	5.0 ± 1.0
			2.5 [0.0–7.5]	10.0 [5.3–12.8]	5.0 [4.0–9.8]	5.0 [4.3–5.8]

*Note*: Results correspond to the quantitative analysis of staining intensity (picrosirius red, alcian blue, type‐V collagen, and decorin), to the area occupied by positively stained fibers (polarized picrosirius red and Verhoeff) or to the number of vessels positive for CD31, SMA, and D240 per mm^2^ of dermis. Results are shown as average ± standard error of the mean followed by the median and quartile range [first and third quartiles].

* *p* values below 0.05 as compared to control native skin are labeled with asterisks.

**FIGURE 7:**
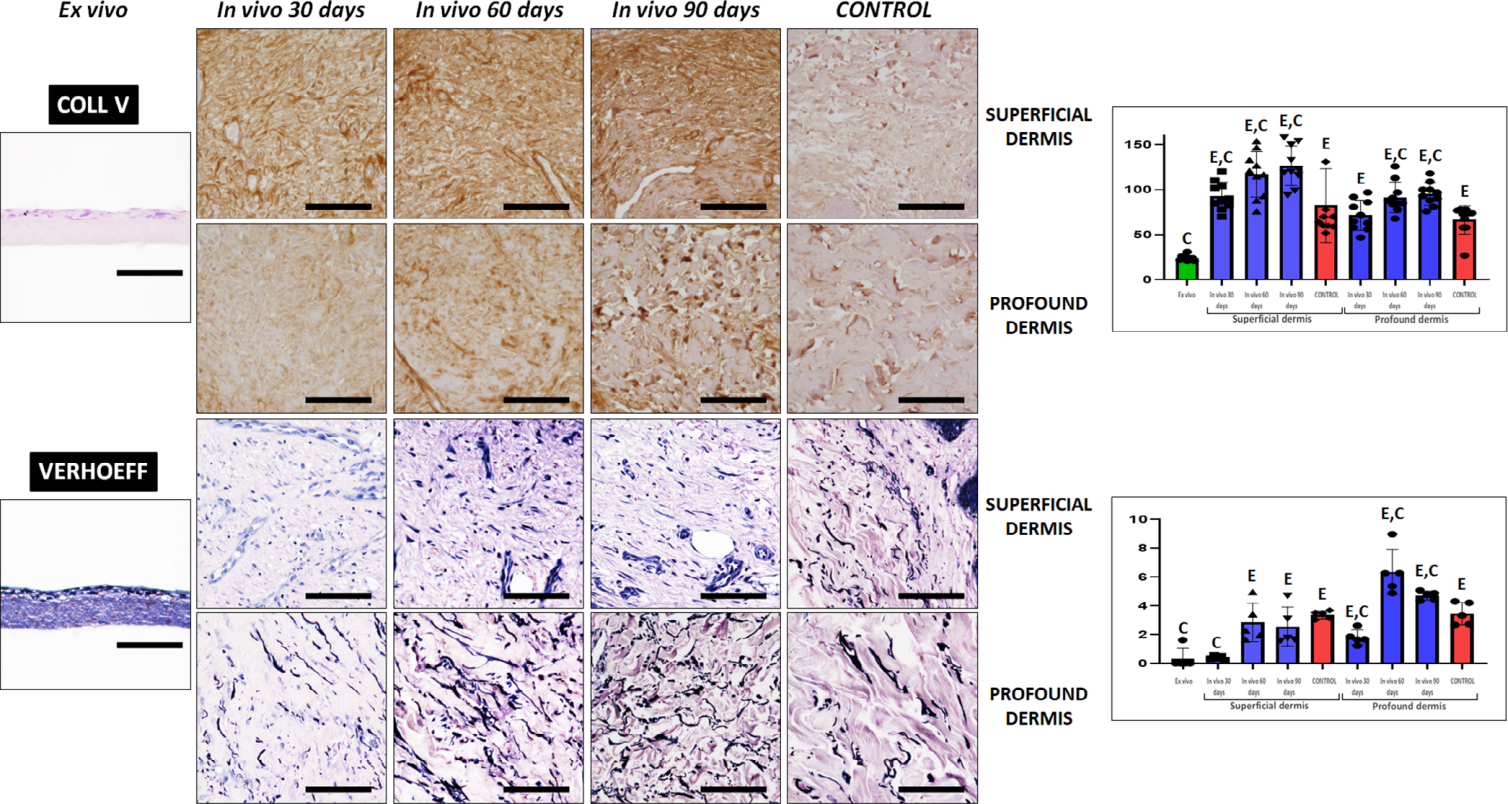
Analysis of type‐V collagen fibers and elastic fibers in control skin and UGRSKIN using immunohistochemistry and Verhoeff histochemical method, respectively. For each type of sample, images were taken at the superficial and profound dermis. Scale bars: 100 μm. Histograms to the right show the average and standard deviation results of the staining signal quantification for each marker expressed as intensity units (I.U.). Significant differences with control native skin are labeled with C, whereas significant differences with samples kept ex vivo are labeled with E. Statistical comparisons were carried out with 10 samples per experimental group.

Then, we analyzed the presence of type‐V collagen by immunohistochemistry (Figure 7 and Tables 2 and 3). Strikingly, expression of this fibrillar component of the extracellular matrix at the superficial layer of the dermis was slight in native skin, and expression was significantly higher in in vivo samples at 30, 60, and 90 days, with ex vivo samples showing significantly lower signal for this protein. When the profound dermis was considered, we found that native skin and biopsies corresponding to 30 days showed similar expression of type‐V collagen than control skin, whilst samples at 60 and 90 days were significantly higher.

Furthermore, Verhoeff histochemistry was used to identify elastic fibers in each type of sample (Figure 7 and Tables 2 and 3). Quantification of the area of dermis occupied by these fibers showed that ex vivo samples and biopsies obtained at day 30 was significantly lower than control native skin at both the superficial and profound dermis, although no differences were found at day 60 and 90.

**FIGURE 8:**
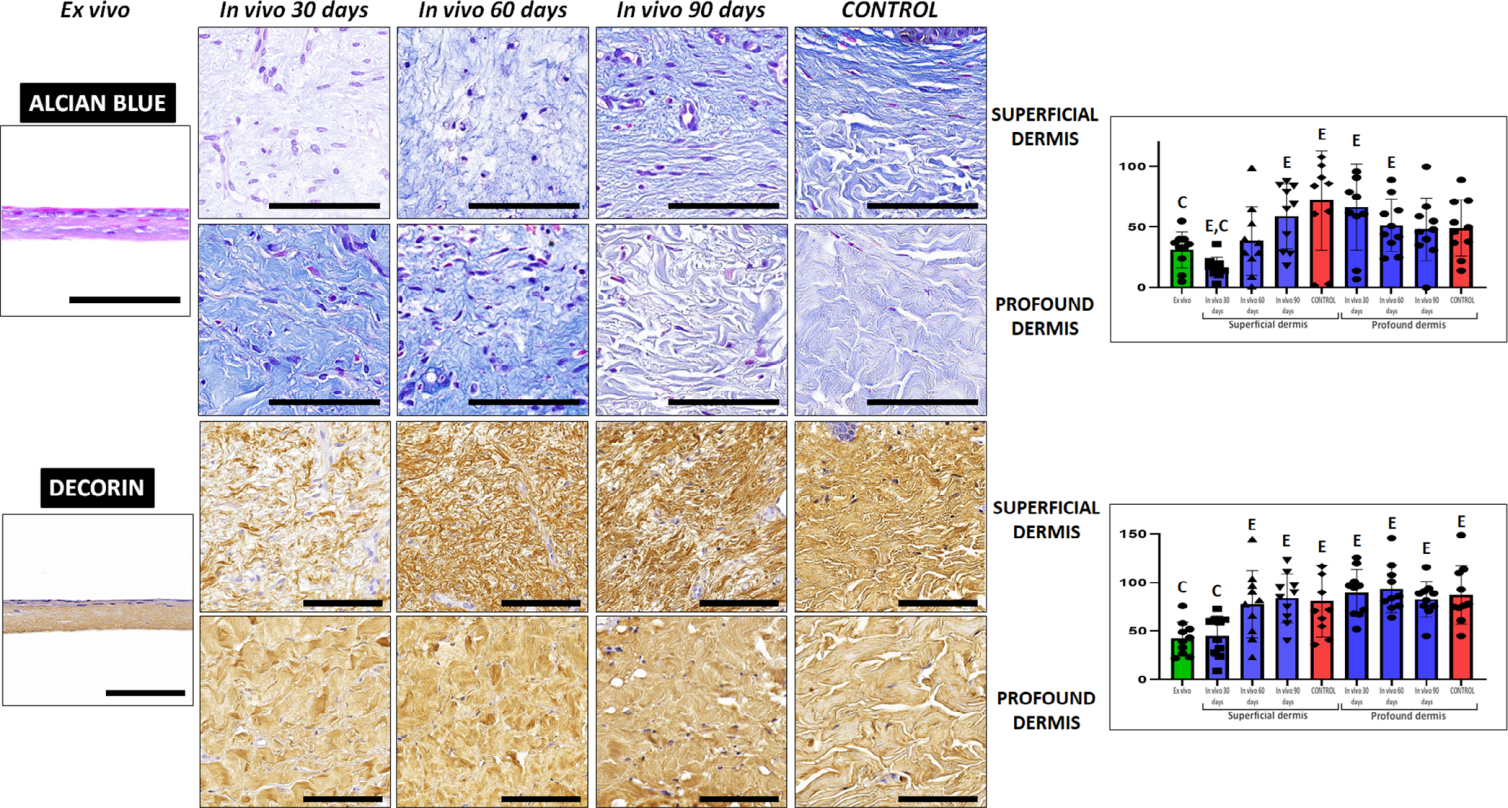
Analysis of non‐fibrillar components of the dermis extracellular matrix using alcian blue histochemistry and decorin immunohistochemistry. For each type of sample, images were taken at the superficial and profound dermis. Scale bars: 100 μm. Histograms to the right show the average and standard deviation results of the staining signal quantification for each marker expressed as intensity units (I.U.). Significant differences with control native skin are labeled with C, whereas significant differences with samples kept ex vivo are labeled with E. Statistical comparisons were carried out with 10 samples per experimental group.

Once the main fibrillar components of the dermis extracellular matrix was evaluated, we analyzed the presence of proteoglycans in each sample type. On the one hand, alcian blue histochemistry revealed that proteoglycans were present in all samples, with the signal intensity found in native skin being similar to the rest of samples, except for the superficial dermis of biopsies at 30 days, which showed significantly lower alcian blue intensity than controls (Figure 8 and Tables 2 and 3). On the other hand, the presence of decorin, a proteoglycan playing a crucial role in dermal matrix assembly, was determined by immunohistochemistry. As shown in Figure 8 and Tables 2 and 3, we found that decorin expression was significantly lower in ex vivo samples and in the superficial dermis of skin grafted for 30 days as compared to control skin dermis. However, decorin expression was comparable to controls for the superficial dermis of 60 and 90‐days biopsies and for the profound dermis of 30, 60, and 90‐days biopsies.

#### 3.3.3 Analysis of blood and lymphatic vessels at the dermal layer

**FIGURE 9:**
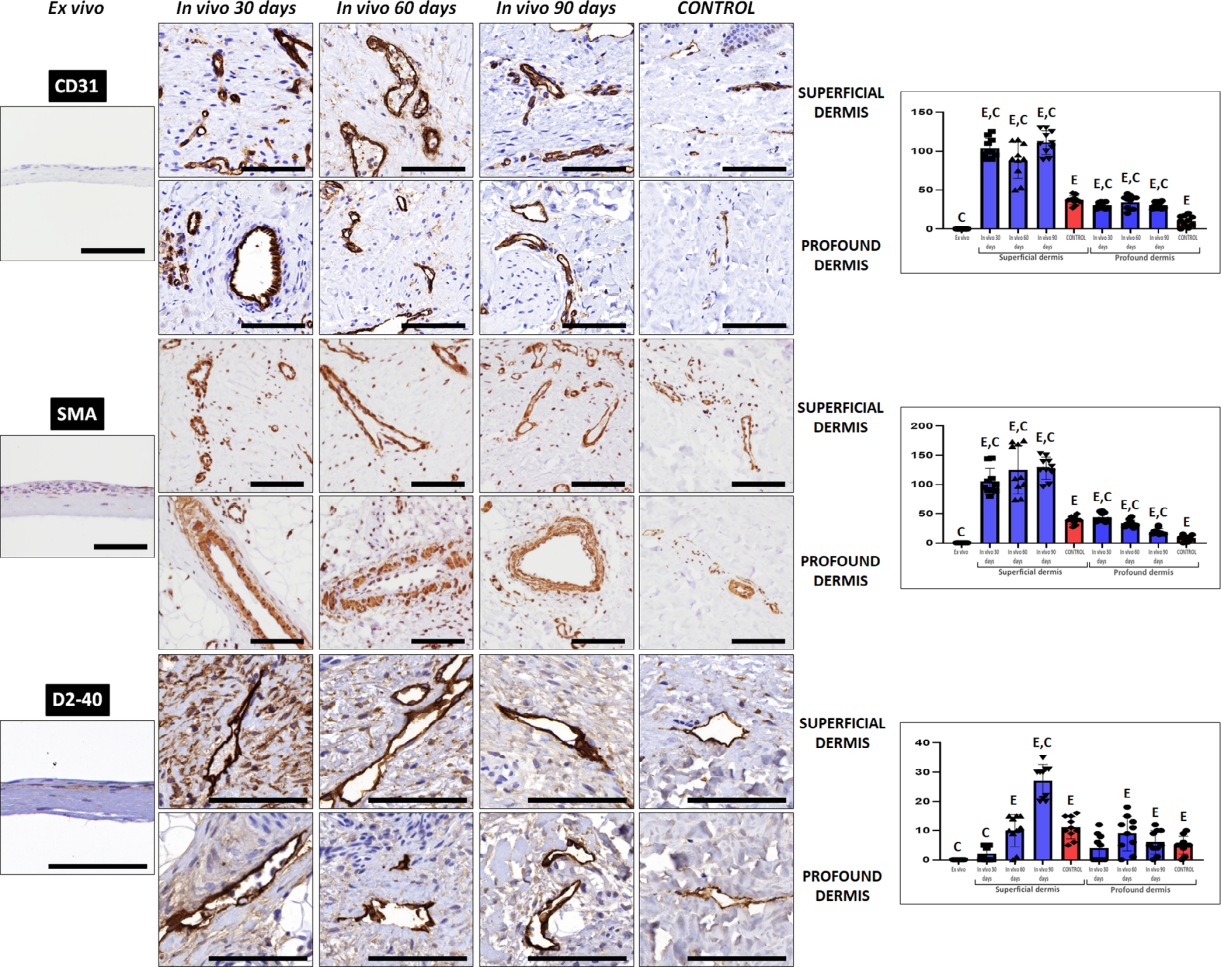
Analysis of blood and lymphatic vessels in the dermal layer of control skin and UGRSKIN using CD31, SMA, and D240 immunohistochemistry. For each type of sample, images were taken at the superficial and profound dermis. Scale bars: 100 μm. Histograms to the right show the average and standard deviation results of the quantitative analysis of vessels showing positive expression of CD31, SMA, and D240 per mm^2^ of superficial and profound dermis Significant differences with control native skin are labeled with C, whereas significant differences with samples kept ex vivo are labeled with E. Statistical comparisons were carried out with 10 samples per experimental group.

In the first place, we analyzed the presence of blood vessels in the dermis of the different samples analyzed in this work using CD31 and SMA immunohistochemistry (Figure 9 and Tables 2 and 3). Results showed that abundant well‐structured blood vessels were found in all samples except for the ex vivo UGRSKIN. Quantification of CD31‐positive vessels revealed that blood vessels were mostly present at the superficial dermis (around 3‐fold as compared to profound dermis) in control skin and in all patient biopsies. Interestingly, we found that biopsies obtained at day 30, 60, and 90 contained a significantly higher number of CD31‐positive structures than control skin in both the superficial and profound dermis. Very similar results were found for SMA‐positive blood vessels. In both cases, the number of blood vessels found at the different days of follow‐up were very similar, with abundant vessels found from day 30 onward.

Regarding lymphatic vessels, our analysis using D240 antibodies revealed that this type of vessel was less abundant in the skin than blood vessels and that the superficial dermis of control skin was more enriched in these structures than the profound dermis (Figure 9 and Tables 2 and 3). At the superficial dermis, quantification of D240‐positive vessels showed that lymphatic vessels were very scarce at day 30 (significantly lower than controls), but their number tended to increase with follow‐up time and became significantly higher than controls at day 90. In contrast, analysis of the profound dermis showed that the number of lymphatic structures was similar to controls at all study times.

## 4 DISCUSSION

Advanced in tissue engineering allowed the development of efficient ATMPs for the treatment of severely burnt patients and contributed to save many lives over the last decades.^21^ In our case, the UGRSKIN model of bioartificial skin demonstrated to be highly biocompatible and contributed to skin regeneration in 75% of the patients.^13^ However, the biological mechanisms associated to its therapeutic effect still need to be elucidated. This information would contribute to a proper characterization of the ATMP as requested by the European regulatory agencies and could establish the bases for a future improvement of the technology to achieve more efficient results.

In the present work, we have carried out a comprehensive structural and ultrastructural characterization of the bioartificial skin grafted in severely burnt patients using an array of histological, histochemical and immunohistochemical methods. To our knowledge, this is one of the first studies using a high number of histological analyses and relevant skin markers at the different layers of a bioartificial skin grafted in patients. Noteworthily, the use of skin biopsies obtained at different follow‐up times from the same patient contributed to control confounding variables such as the age, gender, or disease severity, which may influence the results, as previously reported.^18^

In general, our results revealed that the UGRSKIN model was highly biocompatible and became rapidly integrated at the host tissue from the first biopsies obtained at day 30th, although a complete biointegration required longer time periods. When the epidermal layer was analyzed histologically, we found very few differences with control normal skin at all study times. The fact that the grafted skin had well‐developed basal, spinosum, granulosum and corneum strata from day 30th suggests that the UGRSKIN model, which did not have this level of differentiation ex vivo, was able to integrate and differentiate in response to the in vivo environment of the host tissue. In this regard, it is well known that bioengineered tissues kept in culture are not fully differentiated, and the in vivo setting is able to induce cells to fully develop and differentiate in response to numerous paracrine signals and environmental factors.^15^ Interestingly, the ultrastructural analysis of each epidermal stratum revealed that keratinocytes were fully differentiated and showed typical intercellular junctions and keratohyalin granules and were able to differentiate into keratinized structures at the most apical layers. These findings reveal that the bioengineered epidermis grafted in patients show high similarity with the native skin from the first moment of the analysis onward, suggesting that the skin barrier function of the epidermis, which is greatly dependent on the structure and composition of the epidermis,^22^, ^23^ is restored in the burnt patient.

To determine if the skin keratinocytes expressed crucial proteins of epithelial differentiation, we performed several immunohistochemical analyses of the epidermal layer. First, we found that grafted skin keratinocytes expressed several cytokeratins found in native skin. On the one hand, samples showed positive expression of the keratinization marker CK10, which is in agreement with the keratinization signs found at the morphological level in all samples grafted in vivo, and was negative for CK8, a marker of non‐stratified simple epithelia.^24^, ^25^, ^26^ On the other hand, the grafted skin showed positive expression of the epithelial proliferation marker CK5 and showed a high number of basal cells labeled with anti‐Ki67 antibodies, especially at days 60 and 90 of in vivo development. These results suggest that the epidermis grafted in patients was able to differentiate into a functional epithelium that is very similar to the native skin without losing the proliferative capability of the normal human epidermis. Hence, it has been demonstrated that the regenerative capacity of the human epidermis is strictly dependent on a specific population of basal keratinocytes known as *epidermal proliferative unit* (EPU). These cells proliferate actively in vivo and show positive expression of several markers of cell proliferation.^27^

Then, our analysis of specific cell‐cell junctions proteins confirmed that epidermal keratinocytes were firmly attached by intercellular junctions from day 30th onward, which coincides with the results found by the ultrastructural analysis. Thus, the presence of abundant intercellular desmosomes and tight junctions in a similar way to control skin suggests that the grafted skin could form a functional protective barrier from a very early stage. In agreement with these results, two major markers of epidermal differentiation‐filaggrin and involucrin‐playing a crucial role in the formation of a cornified layer and control of the hydration level of the epidermis^28^ were also found in the analyzed samples from the first analyzed biopsy onward.

All these results reveal that the epidermal layer of the UGRSKIN model grafted in burnt patients was able to differentiate and mature very early, and became comparable to control epidermis in response to the in vivo environment. The fact that the epidermis expressed several key markers of epithelial function and differentiation suggests that the skin barrier function could be fully functional in these patients from day 30th onward. Given that the main role of the human skin is serving as a first line of defense against potentially harmful agents,^29^ formation of a functional barrier from the first days of follow‐up could contribute to explain the very positive clinical results found in patients treated with the UGRSKIN model,^13^ since a prompt reestablishment of the protective barrier of the human skin while minimizing infection, scarring, and contracture is crucial for patient survival.^30^, ^31^ Other authors have used other approaches for the clinical management of severely burnt patients, such as the implant of autologous micrografts, achieving regeneration and complete healing after 80 days.^32^ The application of micrografts have been associated to an improved epithelial regeneration, together with a significant reduction of granulation tissue.^33^ However, the need of using healthy skin obtained from non‐affected areas of the patient skin makes necessary the search of alternative treatments based on biological skin substitutes generated from human cells expanded in culture.

In general, these results are in agreement with previous reports suggesting that different models of bioartificial human skin grafted in patients show high levels of epidermal differentiation and keratinization from the first moment of the analysis‐2 weeks after the implant in one report^34^ and 9 months after the implant in another one.^35^ Along with our results, these previous reports suggest that human skin keratinocytes tend to mature and differentiate very rapidly in vivo when a highly biocompatible bioartificial skin model is applied to the patient.

In addition to keratinocytes, other types of epithelial cells playing an important role in skin epidermis homeostasis are melanocytes, Langerhans cells and other cell types.^36^ Although these cells were not apparently present in the UGRSKIN model kept in culture, we found that the human skin epidermis grafted in burnt patients contained important amounts of melanocytes and Langerhans cells, especially in samples corresponding to 60 and 90 days of follow‐up. However, the number of cells found in the epidermis of the UGRSKIN model grafted in patients was significantly lower than native control skin at all times. This finding is in agreement with previous studies suggesting that different types of human bioengineered skin substitutes grafted in patients contain significantly less melanocytes and Langerhans cells than native skin.^35^, ^37^ Whether or not this shortage of both cell types has consequences on the patient, reveals unanswered. In this regard, specific biofabrication methods have been described for culturing these cell types, and several models of bioartificial skin containing melanocytes have been reported.^37^

One of the most important structures supporting the skin epithelium is the basement membrane. In agreement with the results found at the epidermal level, we found that the basement membrane was well‐differentiated in all analyzed skin biopsies, confirming again that the UGRSKIN model tended to differentiate from day 30th of in vivo development. Interestingly, the dermo‐epidermal junction was flat and devoid of the typical rete ridges and dermal papillae of the native skin. Although these structures play a key role in epidermal adhesion and skin homeostasis,^38^, ^39^ the absence of rete ridges and dermal papillae has been widely described in all kinds of bioengineered skin substitutes grafted in vivo,^35^ and it is known that rete ridges are not reconstituted after full thickness wound healing and scarring in humans.^40^ Most likely, the absence of physical forces and mechanical stretching in healing skin could explain the absence of these structures.^40^ Interestingly, the lack of these dermo‐epidermal specialized structures could explain the paucity of melanocytes in the grafted skin, since it has been recently demonstrated that the human skin melanoblast populations found at the rete ridge are greater than in the flat areas of the skin.^41^

Once the epidermis and basement membrane were analyzed, we carried out specific analyses of the dermal layer. The human skin dermis mainly consists of a dense ECM containing several cell types, especially fibroblasts, and numerous blood and lymphatic vessels, and its function is crucial for the development and function of the suprajacent epidermis.^42^ In a previous work carried out in laboratory animals, we demonstrated that the in vivo environment is able to induce the UGRSKIN model to remodel the ECM structure and composition.^8^ In the present work, we wanted to determine if the same ECM remodeling process found in animals is reproduced in human patients.

First, we found that the implanted dermis became very rapidly integrated with the host tissue, as it was the case of the epidermis. In fact, we did not find any rests of the grafted nanostructured fibrin‐agarose biomaterial at any of the follow‐up periods analyzed here, and the ECM of all biopsies was very similar to the control normal ECM dermis, suggesting an active ECM remodeling and biointegration process. However, two main differences were found as compared to the native skin: the lack of a papillary and a reticular dermis and the presence of a clearly detectable interface between the superficial and the profound dermis in the first biopsies. The absence of a papillary dermis is unsurprising, since the grafted skin is devoid of rete ridges, as discussed above. Regarding the interface, we hypothesize that it may correspond to the boundary between the grafted bioengineered skin and the remaining host tissue, which tended to disappear with time as the biointegration process increases, giving rise to the development of a superficial skin and a profound dermis.

Normal skin ECM is mostly composed of collagen fibers, which account for 75% of the skin and provide strength and elasticity.^42^ Our analysis of collagen fibers as determined by picrosirius red histochemistry revealed that the grafted skin contains substantial amounts of collagen, especially at the profound dermis, although it requires at least 90 days of follow‐up to reach the concentration found in control skin at the superficial dermis layer. Fiber orientation and maturation, which is related to detectable birefringence,^43^ is a major requirement of collagen fibers to exert their biomechanical functions. For this reason, we also quantified the presence of mature, properly‐oriented collagen fibers using polarized light, and we found that the skin biopsies corresponding to 90 days of development were rich in this kind of fibers, similar to control skin at both the superficial and profound dermis. These results suggest that although UGRSKIN is integrated and remodeled at the host site from day 30th, full development and maturation of the dermal collagen fibers might require up to 90 days of follow‐up, and the biomechanical properties of the grafted tissue could be suboptimal until this moment.

Other types of collagen also play a role in the human dermis such as type‐V collagen comprising around 5% of the total collagen content of the normal dermis.^42^ Type‐V collagen fibrils are able to form a network in the human dermis, which is associated to other fibrillar collagens, elastic fibers and proteoglycans and provides structural integrity to the dermis.^44^ While our bioengineered skin model was devoid of type‐V collagen while kept in culture, we found a sequential increase of this component in the dermis of all grafted samples, especially at the superficial dermis, reaching levels that were higher than native human skin. The reasons why the grafted skin became enriched in type‐V collagen remain elusive. However, it has been demonstrated that type‐V collagen acts as a dominant regulator of collagen fibrillogenesis and is overexpressed in tissues synthetizing high amounts of collagen.^45^ The active type‐I collagen biosynthetic process that is active in UGRSKIN samples grafted in patients could be induced by a previous overexpression of type‐V collagen in the same samples. Future studies should determine if this overexpression of type‐V collagen tends to disappear after longer follow‐up periods.

Other important fibrillar components of the human dermis are elastin and elastic fibers, which are able to resist mechanical deformation without altering the normal configuration of the skin^42^ and are directly linked to the mechanical properties of the normal skin. Again, we found that these fibers were present in the grafted skin from the first moments, but grafted skin required up to 60 days to reach the normal levels of native skin, suggesting that the skin implanted in burnt patients could have elastic properties from this moment.

The human skin contains several non‐fibrillar components that are very important for dermal physiology. Among these components, proteoglycans are the most abundant and essential molecules working as major regulators of proliferation, migration, protein synthesis and degradation.^18^ In this respect, we found that the grafted skin showed high amounts of proteoglycans at all times, except for the superficial dermis at 30 days, and the same results were found for decorin. Decorin is one of the most important proteoglycans in the human dermis playing an important role in dermal collagen fibril assembly.^46^ The fact that decorin and proteoglycans in general were found at physiological levels in the dermis of most samples again supports the idea that the UGRSKIN model became biointegrated and was able to synthesize major ECM components very rapidly.

As a connective tissue, the human skin dermis is characterized by the presence of abundant blood vessels providing nutrients and oxygen to skin cells. In fact, angiogenesis is one of the main factors driving in vivo biointegration of artificial tissues, and it has been demonstrated that the lack of vascularization could lead to tissue hypoxia and treatment failure.^47^, ^48^ In our study, we found that the UGRSKIN model grafted in patients developed a very abundant vascular network from day 30th, suggesting that the grafted skin was able to stimulate host blood vessels to grow and extend toward the implanted tissue to achieve biointegration. The presence of CD31 and SMA‐positive structures suggests that the vessels developed by the grafted skin are well‐developed vessels containing both the endothelial layer and the smooth muscle layer of medium and large‐size vessels.^49^ These results are in accordance with previous reports showing that the use of autologous skin micrografts for the treatment of posttraumatic skin defects was associated to an early expression of CD31 and SMA positive vessels, leading to skin regeneration and avoiding the outcome scar.^33^ Although a higher amount of blood vessels was found at the superficial dermis, we found that grafted skin vessels were not organized into upper and lower horizontal plexuses as it is the case of the normal human skin as previously reported.^50^ The rapid biointegration process of the artificial skin and the fact that the host tissue was severely injured could be the responsible of this vessel distribution in the patients' dermis. Although future studies should be carried out in this sense, it is likely that vessels could reorganize and distribute in a different manner at longer follow‐up times. Regarding lymphatic vessels, we found very few differences between the profound dermis of the grafted skin and the native skin. However, the number of lymphatic vessels found in samples grafted for 30 days was significantly lower than controls at the superficial dermis, and this number tended to increase with time. It has been proposed that lymphatic vessels are key actors controlling tissue microenvironment that play a major role in multiple physiological and pathophysiological processes, such as control of tissue hydration and regulation of immune functions.^51^ Therefore, our findings point out that the regenerating skin is also capable of attracting abundant lymphatic vessels from the host tissue in order to ensure a normal physiology of the regenerated skin.

Finally, our analysis of the subcutaneous adipose tissue (hypodermis) showed no differences with control skin, and this structure was formed by adipocytes and are subdivided by multiple septae of connective tissue in all samples, at previously reported for the normal human skin.^52^ Most likely, these findings correspond to profound layers of the patient skin that were not irreversibly burned.

The present study has several limitations. Although UGRSKIN model has demonstrated potential usefulness to regenerate all skin layers, the real regenerative potential and clinical outcomes of patients treated with UGRSKIN should be determined in the future using a larger cohort of patients. In addition, the UGRSKIN model should be improved to generate more biomimetic skin substitutes showing the typical rete ridges, a vascular network and skin appendages to obtain a more biomimetic skin substitute able to reproduce the structure of the native skin. In addition, this skin model is strictly dependent on the availability of skin biopsies obtained from burnt patients, and the time required to generate the UGRSKIN substitute should be shortened in the future. These drawbacks could be surpassed by using alternative cell sources, as previously reported.^15^

In conclusion, our comprehensive histological, histochemical and immunohistochemical characterization of the UGRSKIN bioartificial skin model grafted in patients provides several clues explaining the very good biocompatibility and clinical efficiency of this ATMP used in severely burnt patients. In general, we found that the epithelial layer tends to mature and differentiate very rapidly from the first day of the analysis, and this could be one of the main factors contributing to patient survival. Similarly, the dermal layer demonstrated to be highly biocompatible, but required longer periods of time to fully differentiate and integrate at the host tissue. Future studies should be carried out to determine the stability of the regenerated skin after longer periods of time.

## AUTHOR CONTRIBUTIONS

**Miguel Angel Martin‐Piedra:** Data curation (equal); investigation (equal); writing – original draft (equal); writing – review and editing (equal). **Gloria Carmona:** Data curation (equal); investigation (equal); writing – original draft (equal); writing – review and editing (equal). **Fernando Campos:** Formal analysis (equal); methodology (equal). **Víctor Carriel:** Data curation (equal); formal analysis (equal); methodology (equal). **Ana Fernández‐González:** Formal analysis (equal); methodology (equal). **Antonio Campos:** Conceptualization (equal); funding acquisition (equal); project administration (equal). **Natividad Cuende:** Project administration (equal); validation (equal). **Ingrid Garzón:** Conceptualization (equal); methodology (equal); supervision (equal); validation (equal); writing – review and editing (equal). **Purificación Gacto:** Conceptualization (equal); project administration (equal); resources (equal). **Miguel Alaminos:** Conceptualization (equal); funding acquisition (equal); project administration (equal); supervision (equal); writing – review and editing (equal).

## CONFLICT OF INTEREST STATEMENT

Authors declare that they do not have any conflict of interest.

### PEER REVIEW

The peer review history for this article is available at https://www.webofscience.com/api/gateway/wos/peer-review/10.1002/btm2.10572.

## Supporting information


**Table S1.** Primary antibodies used for immunostaining analysis. ON, overnight hybridization.

## Data Availability

The data that support the findings of this study are available from the corresponding author upon reasonable request.
