# Supplementary material for: Histological assessment of nanostructured fibrin‐agarose skin substitutes grafted in burnt patients. A time‐course study
Source: Bioeng Transl Med. 2023 Jul 7;8(6):e10572. doi: 10.1002/btm2.10572 (PMC10658487; doi:10.1002/btm2.10572)
Supplement: Supplementary file 1 — Table S1. Primary antibodies used for immunostaining analysis. ON, overnight hybridization. [file BTM2-8-e10572-s001.docx]

**Supplementary Table S1**. Primary antibodies used for immunostaining analysis. ON: Overnight hybridization.

| **ANTIBODY** | **HIBRIDIZATION** | **REFERENCE** |
| --- | --- | --- |
| Rabbit anti-Cytokeratin 8 | Prediluted, ON | Master Diagnostica, Granada, Spain, MAD-000693QD |
| Mouse anti-Cytokeratin10 | Prediluted, ON | Master Diagnostica, Granada, Spain, MAD-000150QD |
| Cytokeratin 5 | Prediluted, ON | Master Diagnostica, Granada, Spain, MAD-000491QD |
| Mouse anti-Filaggrin | 1:50, ON | Abcam, Cambridge, UK, ab17808 |
| Mouse anti-Involucrin | Prediluted, ON | Master Diagnostica, Granada, Spain, MAD-000164QD |
| Mouse anti-Claudin 1 | Prediluted, ON | Master Diagnostica, Granada, Spain, MAD-000523QD |
| Mouse anti-Plakoglobin |  | Abcam, Cambridge, UK, ab12083 |
| Mouse anti-Melan-A | Prediluted, ON | Master Diagnostica, Granada, Spain, MAD-001767QD |
| Rabbit anti-CD-1a | Prediluted, ON | Master Diagnostica, Granada, Spain, MAD-000673QD |
| Rabbit anti-Collagen V |  | Abcam, Cambridge, UK, ab7046 |
| Goat anti-Decorin | 1:500, ON | R&D System, AF143 |
| Mouse anti-CD31 | Prediluted, ON | Master Diagnostica, Granada, Spain, MAD-002048QD |
| Mouse anti-D240 | Prediluted, ON | Master Diagnostica, Granada, Spain, MAD-000402QD |
| Mouse anti-SMA | Prediluted, ON | Master Diagnostica, Granada, Spain, MAD-001195QD |
